# Intimate partner violence, behaviours associated with risk of HIV acquisition and condom use in married women in Manicaland, East Zimbabwe: An HIV prevention cascade analysis

**DOI:** 10.1186/s12905-024-03428-x

**Published:** 2024-11-06

**Authors:** Alexandra A. Cordeiro, Louisa Moorhouse, Tawanda Dadirai, Rufurwokuda Maswera, Angela Y. Chang, Constance Nyamukapa, Simon Gregson

**Affiliations:** 1 MRC Centre for Global Infectious Disease Analysis, Department of Infectious Disease Epidemiology, School of Public Health, Imperial College London, Level 2, Faculty Building South Kensington Campus, London, SW7 2AZ UK; 2 Biomedical Research and Training Institute, 10 Seagrave Avondale, Harare, Zimbabwe; 3 Danish Institute for Advanced Study, University of Southern Denmark, Fioniavej 34, Odense, 5230 Denmark; 4 Department of Clinical Research, University of Southern Denmark, J.B. Winsløws Vej 19,3, Odense, 5000 Denmark

**Keywords:** Intimate partner violence, HIV prevention cascade, health education and promotion, community based survey

## Abstract

**Background:**

Intimate partner violence (IPV) is widespread in the WHO African region with generalised HIV epidemics and may contribute to ongoing HIV transmission through its associations with behaviours associated with HIV acquisition risk and low use of prevention methods particularly in marital relationships.

**Methods:**

We conducted a male condom HIV prevention cascade analysis using data from a general-population survey in Manicaland, Zimbabwe (July 2018-December 2019) to develop an understanding of how interventions that reduce IPV might be built upon to also reduce HIV incidence. Multivariable logistic regression was used to measure associations between currently-married HIV-negative women’s experience of IPV and: (1) being in the priority population for HIV prevention methods (i.e. married women engaging in behaviours associated with HIV acquisition risk or with a spouse who engages in similar behaviours or is living with HIV), and (2) male condom use by women in this priority population. Male condom HIV prevention cascades, with explanatory barriers for gaps between successive cascade bars (motivation, access and effective use), were compared for women in the priority population reporting and not reporting IPV.

**Results:**

We found a positive association between IPV and being in the priority population for HIV prevention methods (72.3% *versus* 58.5%; AOR = 2.26, 95% CI:1.74–2.93). Condom use was low (< 15%) for women in the priority population and did not differ between those reporting and not reporting IPV. The HIV prevention cascades for women reporting and not reporting IPV were similar; both showing large gaps in motivation and capacity to use male condoms effectively. Women reporting motivation and access to male condoms were more likely to report their partner being a barrier to condom use if they experienced IPV (84.8% *versus* 75.5%; AOR = 2.25, 95% CI:1.17–4.31).

**Conclusion:**

The findings of this study support the case for trials of integrated IPV/HIV prevention interventions that are tailored to improve HIV risk perception among HIV-negative married women and to make condom provision more acceptable for this group.

**Supplementary Information:**

The online version contains supplementary material available at 10.1186/s12905-024-03428-x.

## Background

Intimate partner violence (IPV) is the most common form of gender-based violence worldwide, a violation of fundamental human rights, and a significant global public health problem [1, 2]. Globally, it is estimated that one-in-three women experience some form of violence perpetrated by a current or former partner at least once during their lifetime [3]. The WHO African region records the highest rates of IPV, with an estimated prevalence of 37%, nearly a quarter more than the global average of 30% [3]. A recent systematic review found that, among ever-partnered women, aged 15–49 years in the African region, lifetime and past-year prevalence of physical violence, sexual violence or both were 27% and 14%, respectively [4]. The high prevalence of IPV in the African region underscores the importance of addressing violence against women; however, progress towards meeting Sustainable Development Goal target 5.2 – to eliminate violence against women and girls by 2030 – has been grossly inadequate [3, 4].

IPV has far-reaching consequences for women’s physical and mental health [5–7]. Importantly, these include its contribution to ongoing HIV transmission. In many African populations, high levels of IPV co-exist with continuing generalised HIV epidemics. In Zimbabwe, among currently-married women living with HIV aged 15–49 years, 34% have experienced physical violence while 13.5% have experienced sexual violence by a current spouse in the past 12 months [8]. In a study in South Africa, IPV was associated with reduced viral suppression in young women on antiretroviral treatment [9].

Furthermore, women experiencing IPV have been found to be 1.5 times more likely to acquire HIV compared to those not affected by IPV [10]. In a national survey in India, currently-married women who experienced IPV were twice as likely to be living with HIV compared to other married women [11]. Some of these associations may be due to direct linkages between IPV and the biological and proximate determinants of HIV acquisition [12]. For example, forced sex can increase women’s susceptibility to HIV infection [13] whilst fear of IPV can restrict use of HIV prevention methods [14, 15]. However, the association could also result from overlapping structural drivers, such as harmful gender norms or unequal gender power dynamics [16, 17], and common individual-level determinants including low education [18], age-disparate relationships [19, 20], and alcohol use [21]. Finally, reverse causality is possible; for example, IPV can result when women are accused of introducing HIV into marriages or in marital disputes that arise when male partners have other sexual partners [22].

The strength and pervasiveness of the association between IPV and HIV risk suggests that interventions to reduce IPV could also be effective in reducing the burden of new HIV infections in women [23]. However, to date, scientific trials of interventions that succeeded in reducing IPV have failed to reduce HIV incidence [24]. Therefore, a fuller understanding of the nature of association between IPV and HIV may be necessary to inform the design of future interventions that are effective in controlling both epidemics. In Zimbabwe, relatively little research has separated out the associations between IPV, behaviours associated with HIV acquisition risk, and effective use of HIV prevention methods amongst women at high risk. For the latter, novel approaches such as description and comparative analysis of HIV prevention cascades for HIV-negative married women at risk may be especially helpful in providing insights into differences in the barriers to use of prevention methods in the presence and absence of exposure to IPV. The HIV prevention cascade framework has been developed as a practical and generic framework, through a series of consultations, and draws from social cognitive theoretical frameworks and wider literature to describe determinants of and barriers to HIV prevention method use. The cascade describes a series of steps (motivation, access and capacity to use a prevention method) taken by an individual to reach the end point of HIV prevention method use, and then associated barriers to each of these steps among those who are lost along the pathway of the cascade [26–28].

In this paper, we aim to contribute to filling these gaps in current understanding of associations between IPV, HIV risk and use of HIV prevention methods among HIV-negative married women, by conducting an HIV prevention cascade analysis using data from a general population survey in Manicaland, east Zimbabwe. The primary objectives of this analysis are to:

1. Measure the association between IPV and being in the priority population for HIV prevention (i.e. HIV-negative women at greatest risk of acquiring HIV infection) [25].
2. Measure the association between IPV and male condom use for HIV-negative women in the priority population; and.
3. Compare and investigate where HIV-negative women who experience IPV drop off in the HIV prevention condom cascade compared to HIV-negative women who do not experience IPV.

A secondary objective of the analysis is to provide context and aid interpretation of the results by measuring the associations between individual- and partner-level characteristics and physical and sexual IPV.

## Methods

### Study setting

This study was conducted in Manicaland, Zimbabwe’s second most populous province, with a population of approximately 1.8 million people [26]. The province is located in the east of the country, about 243 km from the capital city Harare, and approximately 83% of its population reside in rural areas [26]. In 2019, Manicaland recorded the highest proportion of poor households and had poor educational and population health outcomes compared to other provinces in Zimbabwe [27]. In Manicaland, 38.5% of ever-married women, aged 15–49 years, reported having experienced physical and/or sexual violence in their lifetime in a 2015/2016 national survey compared to 35.4% for Zimbabwe as a whole [28]. HIV prevalence has declined in Manicaland since the late 1990s [29] but remains high among married women aged 15–49 years (13.5%) [8, 30].

### Data source: The Manicaland HIV prevention cascade study

We conducted a cross-sectional analysis using data collected between July 2018 and December 2019 as part of the Manicaland HIV Prevention Cascade Study (http://www.manicalandhivproject.org/prevention-cascade.html). In an initial census, households were enumerated across eight study sites representing two urban areas, two small towns, one tea estate, one forestry estate, one roadside settlement, and one subsistence farming area. In the census interviews, data were collected on household characteristics, and women aged 15–24 years, males aged 15–29 years, and two-thirds random samples of older males and females (30 + years and 25 + years, respectively) resident in the households were invited to participate in individual interviews. In the individual interviews, data were collected on sociodemographic characteristics, sexual behaviours, HIV status, knowledge, beliefs and perceptions of HIV/AIDS, and use of HIV prevention methods. The study questionnaire included standard UNAIDS questions on IPV [34] (Table 1).

**Table 1:** Measurement definitions for intimate partner violence (IPV) variables

Form(s) of IPV		Survey question* (Have you experienced any of the following from a male intimate partner in the past 12 months? ):						
Physical violence only			1. Slapped you or thew something at you that could hurt you					
			2. Pushed or shoved you					
			3. Hit you with a fist or something else that could hurt you					
			4. Kicked or dragged you or beat you up					
			5. Choked or burnt you					
			6. Threatened or used a gun, knife or other weapon against you					
			Participants were classified as experiencing any form of physical					
			violence (only) if they provided an affirmative response to one or more					
			of the above questions, but to none of the sexual violence questions					
			below.					
Sexual violence only			1. Physically forced you to have sexual intercourse against your will					
			2. Forced you to do something sexual degrading or humiliating					
			3. Made you afraid of what would happen if you did not have sexual					
			intercourse					
			Participants were classified as experiencing any form of sexual					
			violence (only) if they provided an affirmative response to one or more					
			of the above questions, but to none of the physical violence questions					
			above.					
Physical and sexual			If participants answered ‘Yes’ to any of the physical violence AND to					
violence			any of the sexual violence questions, they were classified as					
			experiencing both physical and sexual violence.					

*Data were collected from all female participants who consented to participate in the survey and were willing to answer these questions.

HIV infection status was assessed for survey respondents by provider-initiated testing and counselling (PITC) using the Zimbabwe Ministry of Health and Child Care’s national algorithm [31]. Individuals who declined PITC were requested to provide a dried blood spot (DBS) which was tested at an accredited laboratory (https://brti.co.zw/serology/) using the same algorithm.

Analyses for this study were restricted to currently married/cohabiting women aged 15–54 years for whom a confirmed HIV-negative status was determined and who self-reported being sexually-active in the last twelve months.

### Variables and measures

#### IPV variables

Three mutually-exclusive categories of IPV were defined as follows:

- Experience of any physical violence (only) but not sexual violence by a current or recent partner in the last 12 months;
- Experience of any sexual violence (only) but not physical violence by a current or recent partner in the last 12 months; and.
- Experience of physical and sexual violence by a current or recent partner in the last 12 months.

The UNAIDS questions [34] used to measure experience of physical or sexual IPV (or both) in the past 12-months as shown in Table 1. Binary response categories (“Yes/No”) were used to capture women’s experience of physical, sexual, and physical and sexual violence in the past year.

#### Explanatory variables for experience of IPV

Explanatory variables included the following women’s sociodemographic characteristics: age, level of education (no education, primary, secondary, and higher), occupation type, religion, and place of residence. A household wealth index was estimated from the data on household characteristics and was arranged in terciles (poorest, poor, least poor) [32]. Partner-related explanatory variables included: partner’s age, level of education, and occupation type. Measurement definitions of women’s and partner’s characteristics are described in Table S1.

#### HIV risk behaviours

Risk behaviour variables for HIV acquisition included: alcohol consumption, age at first sex, two or more sexual partners in the last 12 months, one or more non-regular partners in the last 12 months, ever engaged in transactional sex, partner has a sexually transmitted infection (including HIV), and partner has other sexual partner(s). Measurement definitions of HIV risk-behaviours are described in Table S2.

#### HIV prevention cascade variables

Data on the three main bars of a generic HIV prevention cascade (motivation, access, and effective use) [33], measured here for male condoms, and on the corresponding explanatory barriers (sub-bars) were collected in the individual questionnaire. Definitions for the main bars and the sub-bars in the generic cascade were as described by Moorhouse and colleagues [34].

### Statistical analysis

Proportions and 95% confidence intervals (CIs) for women reporting IPV (physical violence only, sexual violence only, and physical and sexual violence) were calculated for all women and by women’s and partner’s sociodemographic characteristics. Multivariable logistic regression measured the associations between: (i) women’s sociodemographic characteristics and exposure to the different forms of IPV; and (ii) partners’ sociodemographic characteristics and exposure to IPV; first adjusting only for women’s age and then adjusting for all independent variables. A p-value of *p* < 0.2 was used as cut-off for inclusion in the fully-adjusted models.

Prevalence estimates of each high-risk behaviour for HIV acquisition were calculated for women exposed and not exposed to each form of IPV. Multivariable logistic regression was used to estimate associations between women’s experiences of IPV and engagement in each HIV risk-behaviour adjusted for age and study site.

Four alternative definitions of priority populations who could benefit from HIV prevention methods were considered: (1) women with multiple sexual partners and/or at least one non-regular partner in the last 12 months; (2) women meeting the first definition plus those who started sex before age 17 or who drink alcohol; (3) women meeting the second definition plus those with a regular partner with HIV or another STI; and (4) women meeting the third definition plus those with a regular partner who has other sexual partners. Multivariable logistic regression was used to estimate the age- and study site-adjusted associations between women’s experience of IPV and: (1) being included in the priority population for HIV prevention under these four different definitions; and (2) for condom use amongst women in the priority population under the fourth definition.

HIV prevention cascades for male condoms were populated and compared for women in the priority population under the fourth definition among women experiencing IPV and women not experiencing IPV (Table 2). In constructing the HIV prevention cascades, it was assumed that participants who reported using male condoms effectively were motivated to use and had access to the prevention method [34]. The condom cascade was constructed as a conditional cascade, whereby each step along the cascade was conditional on the preceding step. The condom HIV prevention cascades for married women experiencing and not experiencing IPV were compared to identify whether there were differences in the gaps and explanatory barriers in the cascade that might indicate requirements for different interventions for those experiencing IPV. Exact definitions are described in Table S3. Associations between experiencing IPV and the main and explanatory cascade bars were assessed using logistic regression. Visualization of the condom cascades was carried out in Tableau desktop [35]. Proportions and 95% CIs of the main bars were calculated. Logistic regression models, adjusted for 5-year age group, were used to test for differences between the main and explanatory bars in the condom cascades for women experiencing and not experiencing IPV.

**Table 2:** Percentages of married women experiencing intimate partner violence included in priority populations for HIV prevention based on different combinations of behaviours associated with HIV acquisition risk and associations between intimate partner violence and inclusion in priority populations for HIV prevention

Intimate partner violence					Multiple partners or non-regular partners (PP1)				Early sexual debut (< 17 yrs), alcohol Use, or PP1 (PP2)				Marital partner with an STI (including HIV) or PP2 (PP3)				Marital partner with other partners or PP3 (PP4)					
exposures																						
															AOR 95% C.I.							
					%		AOR 95% C.I.		%		AOR 95% C.I.		%		AOR 95% C.I.		%		AOR 95% C.I.		N	
Physical violence (only)					4.7		1.38 (0.75–2.53)		26.9		1.59 (1.19–2.12)		31.1		1.60 (1.21–2.11)		70.0		1.98 (1.48–2.65)		301	
Sexual violence (only)					7.5		2.50 (0.73–8.58)		20.6		1.12 (0.53–2.37)		28.0		1.36 (0.69–2.70)		71.6		2.43 (1.07–5.56)		41	
Physical and sexual violence					8.0		2.56 (1.12–5.85)		43.3		3.23 (2.03–5.14)		48.7		3.21 (2.03–5.08)		80.4		3.67 (2.00-6.74)		86	
No intimate partner violence					3.1		1		18.3		1		21.9		1		58.5		1		2034	
Physical or sexual violence					5.7		1.69 (1.04–2.77)		29.6		1.81 (1.42–2.31)		34.4		1.83 (1.45–2.32)		72.3		2.26 (1.74–2.93)		428	
No intimate partner violence					3.1		1		18.3		1		21.9		1		58.5		1		2034	
N					92				516				603				1449				2462	

AOR: age- and study site-adjusted odds ratios for being in the priority population for HIV prevention methods compared to women reporting no form of intimate partner violence.

PP1, PP2, PP3 and PP4: priority populations for HIV prevention based on woman’s and her marital partner’s behaviours associated with HIV acquisition risk. Percentages of women experiencing intimate partner violence included in priority populations for HIV prevention weighted to account for over-sampling of males aged 15-29 years and females aged 15-24 years in the survey.

Statistical analyses were performed in R (version 1.1.463) and STATA (version 17). Results from statistical tests were considered statistically significant when *p* < 0.05.

## Results

### Characteristics of married women in Manicaland, east Zimbabwe

The survey participation rate was 77.5% (9802/12651) with 2458 currently married/cohabiting sexually active HIV-negative women aged 15–54 years meeting the inclusion criteria for analysis (Figure S1). The sociodemographic characteristics of the study population are described in Table 3.

**Table 3:** Association between women’s and partners sociodemographic characteristics and experience of IPV in the last 12 months, in HIV-negative married women (15–54 years) in Manicaland, east Zimbabwe, 2018–2019

Explanatory variables			Physical violence (only)				Sexual violence (only)				Physical and sexual violence						
			%	AOR^a^ 95% C.I.	p-value		%	AOR^a^ 95% C.I.	p-value		%	AOR^a^ 95% C.I.	p-value		N		
*Women’s characteristics*																	
Age (years)																	
	15–24		18.1	1			5.6	1			3.8	1			852		
	25–34		16.4	1.08 (0.79–1.48)	0.638		4.6	0.88 (0.51–1.54)	0.663		3.2	1.03 (0.76–1.40)	0.866		776		
	35–44		14.4	1.13 (0.71–1.81)	0.605		5.5	1.27 (0.60–2.67)	0.533		3.7	1.10 (0.71–1.73)	0.665		562		
	45–54		8.9	0.62 (0.32–1.23)	0.170		4.4	1.11 (0.38–3.23)	0.852		2.6	0.65 (0.34–1.24)	0.191		268		
Education																	
	Primary		17.8	1			6.0	1			4.8	1			416		
	Secondary		15.9	0.93 (0.68–1.26)	0.625		4.9	0.90 (0.54–1.51)	0.699		3.1	0.96 (0.71–1.30)	0.784		1879		
	Higher		4.4	0.35 (0.13–0.90)	0.029		1.5	0.40 (0.08–1.99)	0.265		1.5	0.29 (0.11–0.74)	0.010		136		
	No education		29.6	1.25 (0.43–3.59)	0.682		22.2	4.09 (1.32–12.7)	0.015		18.5	1.47 (0.55–3.96)	0.446		27		
Employment status																	
	Unemployed		17.2	1			4.9	1			3.5	1			1580		
	Professional/skilled		8.8	1.02 (0.53–1.96)	0.951		1.4	0.41 (0.09–1.79)	0.239		0.0	1.10 (0.59–2.03)	0.770		147		
	Self-employed		16.7	1.46 (0.30–7.18)	0.640		0.0	-	0.977		0.0	1.35 (0.28–6.57)	0.713		12		
	Unskilled/manual labour		18.2	1.27 (0.70–2.31)	0.425		8.0	1.30 (0.52–3.25)	0.576		6.8	1.15 (0.64–2.08)	0.638		88		
	Informal		16.1	1.04 (0.75–1.43)	0.825		6.7	1.53 (0.93–2.50)	0.091		4.5	1.08 (0.79–1.77)	0.630		403		
	Student		6.3	0.46 (0.10–2.01)	0.300		6.3	1.80 (0.40–8.16)	0.444		3.1	1.14 (0.14–9.39)	0.502		32		
	Other		8.7	0.63 (0.37–1.08)	0.091		5.6	1.36 (0.69–2.69)	0.381		2.6	0.80 (0.50–1.28)	0.345		196		
Place of residence																	
	Rural		14.8	1			3.3	1			2.1	1			331		
	Roadside settlement		17.2	1.24 (0.82–1.87)	0.309		3.8	1.08 (0.48–2.42)	0.849		2.8	1.20 (0.81–1.79)	0.365		418		
	Tea/forestry estate		15.8	1.19 (0.78–1.81)	0.422		6.7	1.62 (0.77–3.40)	0.204		4.9	1.18 (0.79–1.77)	0.421		581		
	Town		11.1	0.81 (0.54–1.23)	0.332		3.7	1.01 (0.47–2.17)	0.974		2.1	0.84 (0.56–1.25)	0.380		674		
	Urban		21.6	1.86 (1.17–2.95)	0.009		7.7	2.17 (0.97–4.89)	0.060		5.1	1.90 (1.22–2.96)	0.005		454		
Religion																	
	Christian		14.8	1			4.2	1			2.9	1			886		
	Traditional		26.0	2.11 (0.62–7.13)	0.231		13.3	3.28 (0.69–15.7)	0.137		6.7	2.57 (0.82–8.07)	0.164		15		
	Spiritualist		15.3	0.93 (0.69–1.24)	0.618		5.5	1.14 (0.70–1.85)	0.598		3.2	1.02 (0.77–1.35)	0.889		694		
	Other		16.2	1.03 (0.78–1.36)	0.820		5.4	1.18 (0.74–1.88)	0.474		4.0	1.05 (0.81–1.37)	0.708		809		
	No religion		25.9	1.76 (0.91–3.44)	0.095		9.3	1.67 (0.55–5.04)	0.363		7.4	1.65 (0.85–3.20)	0.136		54		
Household wealth index^b^																	
	Poorest		21.4	1			2.8	1			2.3	1			215		
	2nd poorest		15.0	0.68 (0.46-1.00)	0.051		5.1	1.71 (0.69–4.23)	0.248		3.5	0.74 (0.50–1.08)	0.119		1024		
	3rd poorest		16.8	0.76 (0.49–1.18)	0.215		6.2	2.01 (0.77–5.22)	0.153		4.4	0.83 (0.54–1.28)	0.393		611		
	4th poorest		14.4	0.61 (0.37–1.01)	0.054		5.1	1.47 (0.53–4.15)	0.460		3.0	0.69 (0.43–1.12)	0.137		570		
	Least poor		2.8	0.15 (0.02–1.16)	0.070		2.8	1.34 (0.15–12.2)	0.798		0.0	0.30 (0.07–1.36)	0.118		36		
*Partner’s characteristics*																	
Age (years)^c^																	
	15–24		14.8	0.71 (0.47–1.08)	0.108		4.7	0.84 (0.41–1.70)	0.622		2.5	0.74 (0.50–1.11)	0.144		236		
	25–34		19.7	1			5.3	1			4.0	1			907		
	35–44		14.3	0.68 (0.48–0.96)	0.027		5.7	1.10 (0.62–1.95)	0.739		3.6	0.75 (0.54–1.05)	0.094		698		
	45+		11.3	0.54 (0.33–0.90)	0.017		3.9	0.55 (0.24–1.28)	0.167		2.5	0.57 (0.35–0.93)	0.023		595		
Education																	
	Primary		20.4	1			6.4	1			5.2	1			250		
	Secondary		15.7	0.63 (0.44–0.92)	0.016		5.0	0.79 (0.43–1.48)	0.464		3.3	0.67 (0.47–0.96)	0.029		1991		
	Higher		7.8	0.46 (0.23–0.91)	0.025		3.1	0.69 (0.23–2.02)	0.494		1.6	0.52 (0.28–0.99)	0.048		192		
	No education		36.8	2.08 (0.64–6.73)	0.224		21.1	3.52 (0.83–14.9)	0.464		21.1	1.89 (0.58–6.09)	0.288		19		
Employment status																	
	Unemployed		19.8	1			4.9	1			3.0	1			494		
	Professional/skilled		13.6	0.72 (0.52-1.00)	0.049		5.1	1.02 (0.59–1.79)	0.932		3.7	0.69 (0.50–0.94)	0.020		900		
	Self-employed		16.0	0.69 (0.44–1.10)	0.123		2.8	0.49 (0.19–1.25)	0.136		1.9	0.64 (0.41-1.00)	0.052		213		
	Unskilled/manual labour		15.7	0.63 (0.46–0.88)	0.006		5.8	0.78 (0.44–1.37)	0.385		3.9	0.64 (0.47–0.87)	0.005		778		
	Informal		14.8	0.67 (0.30–1.50)	0.326		7.4	1.56 (0.49–4.94)	0.448		5.6	0.68 (0.31–1.46)	0.321		54		
	Student		15.4	0.72 (0.15–3.42)	0.684		7.7	1.43 (0.17–12.2)	0.745		0.0	-			13		

Cases included in the analysis (N) = 2458.

^a^AOR: Odds ratios from logistic regression models adjusted for all other explanatory variables.

^b^The data needed to calculate the household wealth index was missing for 2 households.

^c^22 women did not know their partners age at his last birthday.

### Levels and patterns of IPV in the last year

**Fig. 1:**
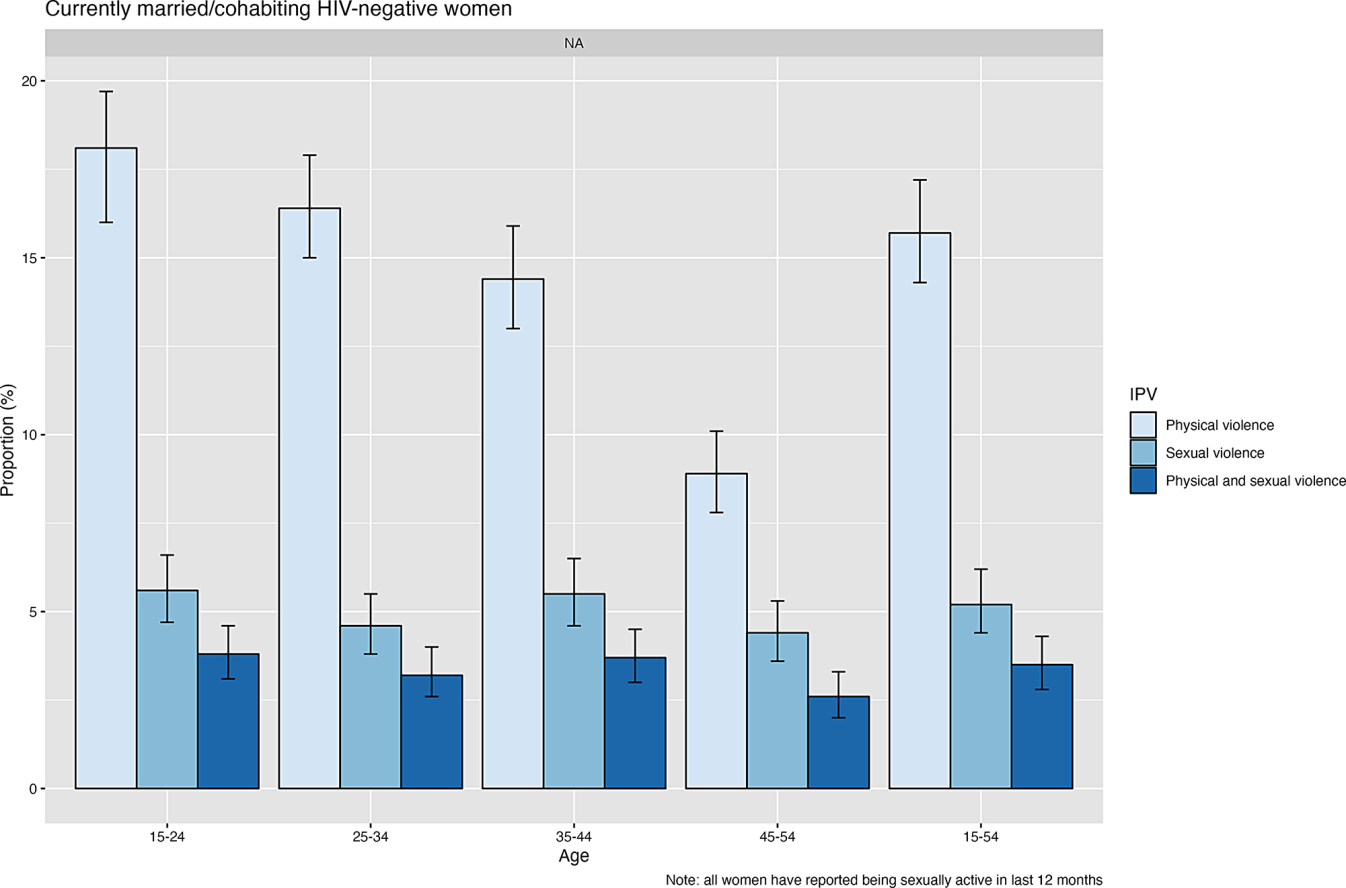
Prevalence of intimate partner violence (physical only, sexual only, and physical and sexual violence) in the last 12 months, reported by currently married/cohabiting HIV-negative women (15–54 years) in Manicaland, east Zimbabwe, 2018–2019

Physical violence (only), sexual violence (only), and both physical and sexual violence were reported by 15.7%, 5.2% and 3.5% of the HIV-negative married women, respectively (Fig. 1).

In univariate analysis, younger age was associated with physical IPV but not with sexual IPV. Women aged 45–54 years had 0.45 times the odds (8.9% vs. 18%; 95% CI: 0.28–0.70; *p* < 0.001) of experiencing physical violence and 0.72 times the odds (4.4% vs. 4.6%; 95% CI: 0.37–1.40; *p* = 0.33) of experiencing sexual violence compared to women aged 15–24 years. In age-adjusted logistic regression analysis, factors associated with IPV were lower education (for all forms of IPV), unemployment and low-skilled and informal sector employment, urban and agricultural estate residence, and no religion (Table S4).

Following full adjustment for women’s sociodemographic covariates, urban residence and lower education still showed statistically significant positive associations with IPV (Table S5). Women living in urban areas had 1.73 times (21.6% vs. 14.8%; 95% CI: 1.11–2.73; *p* = 0.017) and 1.76 times (5.1% vs. 2.1%; 95% CI: 1.15–2.74; *p* = 0.011) the odds of experiencing physical violence and both physical and sexual violence, respectively, of those living in rural areas. Women with higher education had 0.26 times (4.4% vs. 17.7%; 95% CI: 0.10–0.61; *p* = 0.004) and 0.23 times (1.5% vs. 4.8%; 95% CI: 0.08–0.53; *p* = 0.002) the odds of experiencing physical violence and both physical and sexual violence, respectively, of those with only primary school education.

When women’s marital partners’ characteristics are also included in the models, the associations with urban residence and women’s education remained (Table 3). In addition, women with partners aged 25–34 years had higher odds of experiencing physical violence (11.3% in women with partners aged over 45 years vs. 19.7%; AOR = 0.54; 95% CI: 0.33–0.90; *p* = 0.014) and both physical and sexual violence than women with older partners (2.5% in women with partners aged over 45 years vs. 4.0%; AOR = 0.57; 95% CI: 0.35–0.93; *p* = 0.023). Protective associations against physical violence and both physical and sexual violence were found for women whose partners had secondary education (15.7% vs. 20.4% for partners with primary education; AOR = 0.63; 95% CI: 0.44–0.92; *p* = 0.016; 3.3% vs. 5.2%; AOR = 0.67; 95% CI: 0.47–0.96; *p* = 0.029) and higher education (7.8% vs. 20.4%; AOR = 0.46; 95% CI: 0.23–0.91; *p* = 0.016; 1.6% vs. 5.2%; AOR = 0.52; 95% CI: 0.28–0.99; *p* = 0.048) and for women who had partners in formal sector employment (Table 3).

### Association between IPV and being in the priority population for HIV prevention

The results on age- and study site-adjusted associations between married women’s exposure to IPV and engagement in high-risk behaviors for HIV acquisition are shown in Table 4. Women who reported any form of IPV were more likely than other women to report all of the different behaviors associated with HIV acquisition risk except having non-regular sexual partners (which showed a non-significant positive association (*p* = 0.08)) and having a spouse with HIV infection. Women experiencing physical, sexual, and both types of violence all had increased odds of reporting having a spouse with other sexual partners. Women reporting physical violence had greater odds of having started sex before age 17 and multiple sexual partners. Women reporting both types of violence had higher odds of having started sex before age 17, drinking alcohol, and being married to a spouse with a sexually transmitted infection. No statistically significant associations were found between any forms of IPV and having a spouse with HIV infection.

**Table 4:** Associations between physical and sexual intimate partner violence in the last 12 months and HIV risk behaviours in HIV-negative married women in Manicaland, east Zimbabwe, 2018-19

Behaviours associated with HIV acquisition risk				Physical violence (only)			Sexual violence (only)			Physical and sexual violence			Physical or sexual violence			No IPV
				%	AOR 95% C.I.		%	AOR 95% C.I.		%	AOR 95% C.I.		%	AOR 95% C.I.		%
Multiple sexual partners				2.17	2.93 (1.09–7.84)		0.00	-		2.21	3.18 (0.66–15.2)		1.97	2.68 (1.10–6.56)		0.66
Non-regular sexual partner(s)				3.96	1.26 (0.66–2.42)		7.48	2.78 (0.80–9.59)		6.64	2.32 (0.96–5.60)		4.84	1.59 (0.95–2.67)		2.84
Age at first sex < 17 yrs				22.0	1.63 (1.20–2.22)		15.9	1.07 (0.47–2.45)		39.3	3.61 (2.22–5.86)		24.9	1.90 (1.46–2.47)		14.6
Alcohol consumption				2.81	2.10 (0.91–4.82)		0.00	-		4.42	3.47 (1.11–10.8)		2.87	2.14 (1.04–4.38)		1.31
Spouse living with HIV				2.56	0.90 (0.40–2.03)		7.48	2.56 (0.75–8.80)		1.33	0.42 (0.06–3.09)		2.78	0.96 (0.49–1.86)		0.33
Spouse with other STIs				2.43	1.65 (0.71–2.87)		5.61	4.05 (0.93–17.6)		6.64	4.55 (1.71–12.1)		3.59	2.46 (1.28–4.74)		1.48
Spouse has other partners				56.5	1.92 (1.44–2.57)		58.8	2.61 (1.07–6.37)		67.7	3.66 (2.04–6.59)		59.1	2.24 (1.73–2.89)		45.9
N				301			41			86			428			2034

AOR: Age- and study site-adjusted odds ratios for behaviours associated with HIV acquisition risk in married women experiencing intimate partner violence compared to women reporting no form of intimate partner violence

STIs: sexually transmitted infections

Table 4 displays the results on associations between IPV and being in the priority population for HIV prevention under the four alternative definitions. Women who reported ≥ 1 form of IPV and who reported both physical and sexual violence were more likely than women who did not report IPV to be in the priority population for HIV prevention under all four definitions. Women who reported physical violence (only) were not more likely to be in the priority population under the first/narrowest definition but had higher odds than women not reporting IPV after additional risk-behaviours were added (priority populations 2–4). Women reporting sexual violence (only) were only in the priority population for HIV prevention under the broadest definition. Women who reported ≥ 1 form of IPV had twice the odds of being in the fourth priority population (72.3% vs. 58.5%; AOR = 2.26; 95% CI: 1.74–2.93; *p* < 0.001).

### Comparison of male condom use and condom HIV prevention cascades for married women reporting and not reporting IPV

No difference was found in male condom use at last sex between married women in the (fourth) priority population for using an HIV prevention method experiencing and not experiencing any form of IPV after adjusting for 5-year age group (10.0% vs. 8.36%; AOR = 1.27; 95% CI: 0.82–1.96; *p* = 0.28).

**Fig. 2:**
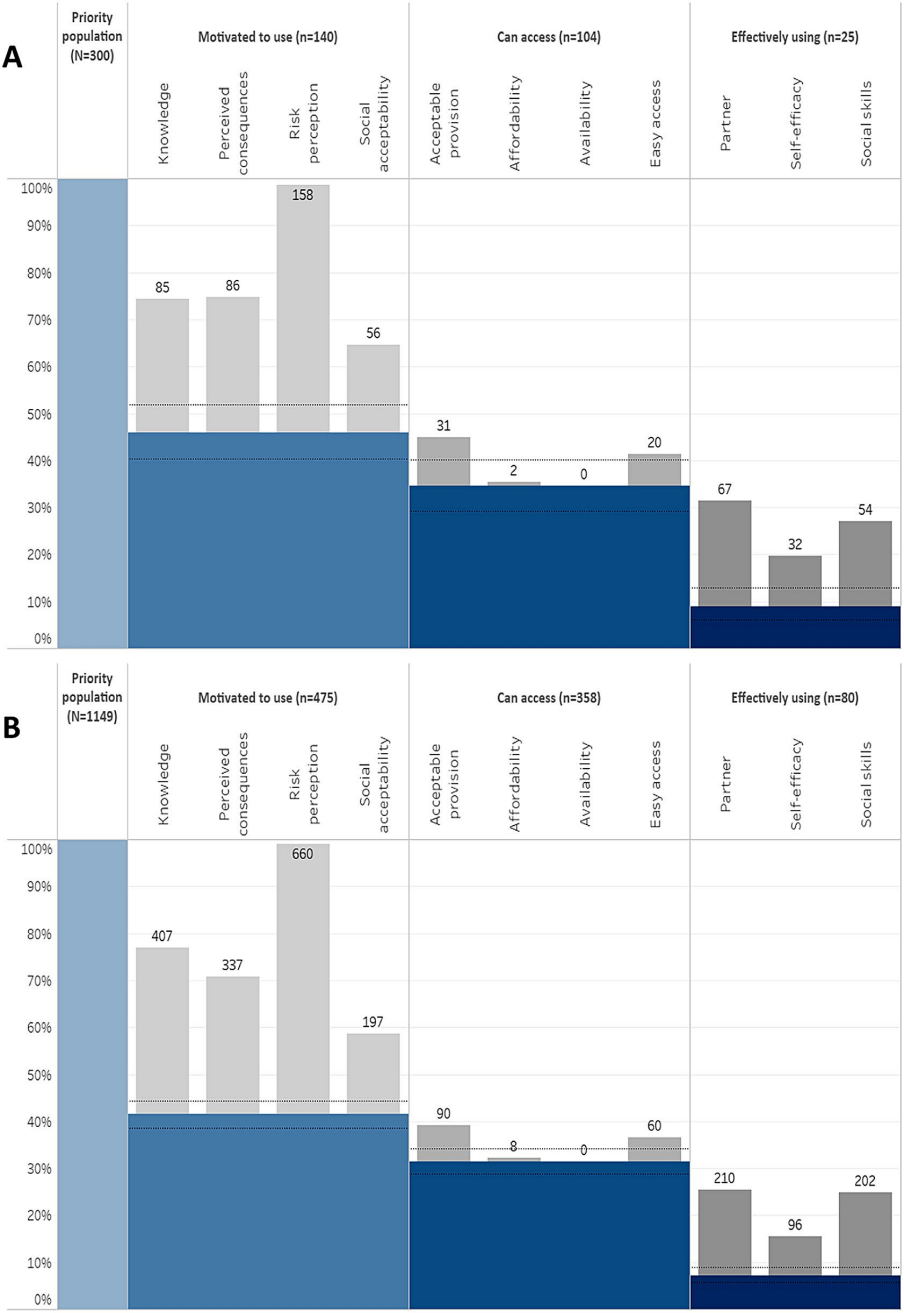
HIV prevention condom cascade for HIV-negative women (15–54 years) who self-reported engaging in at least one risk behaviour for HIV acquisition and (**A**) experiencing IPV; and (**B**) not experiencing IPV. The blue main bars indicate the numbers of individuals from the priority population that are retained at each step of the cascade (shown numerically at the top of the diagram). The grey sub-bars at each step indicate individuals that are lost from the cascade due to the barriers given in the sub-headings to the diagram. Individuals lost from the cascade due to lack of motivation can report more than one barrier to motivation; and similarly for lack of access and lack of effective use

Figure 2 shows the HIV prevention cascades for married women in the priority population experiencing and not experiencing any form of IPV. Of the women experiencing IPV, nearly half (46.7%) were motivated to use condoms. Of the women that were motivated, 74.3% had access to male condoms; however, only 24.0% of those who were motivated and had access were effectively using male condoms (Fig. 2A). Of the women who lacked motivation, almost all (98.8%) did not perceive themselves at risk of HIV, 53.1% had limited knowledge about condoms as an HIV prevention method, and 53.8% perceived negative consequences (e.g. lack of sexual pleasure). 86.1% and 55.6% of women who were motivated to use condoms but lacked access reported lack of acceptable provision and lack of easy access, respectively. Of the women who were motivated and had access to condoms but were not using them, 84.8% reported their partner as a barrier, and 68.4% and 40.5% lacked social (negotiating) skills and self-efficacy, to use condoms.

Within the priority population of married women not experiencing IPV, 41.3% were motivated to use male condoms, 76.9% of these reported having access to condoms, and 22.3% of those who were motivated and had access reported effectively using condoms (Fig. 2B). Of the women who lacked motivation, almost all (97.9%) perceived no risk of HIV, 60.4% had limited knowledge about condoms for HIV prevention, and 50.0% perceived negative consequences. 76.9% and 51.3% of women who were motivated to use condoms but lacked access reported lack of acceptable provision and lack of easy access, respectively. Of the women who were motivated and had access to condoms but were not using the method, 75.5% reported their partner as a barrier. Women in the priority population for HIV prevention therefore were more likely to report lack of partner support as a barrier if they were experiencing IPV than if they were not experiencing IPV (AOR = 2.25, 95% CI: 1.17–4.31; *p* = 0.015). 72.7% and 34.5% of married women not experiencing IPV lacked social skills and self-efficacy to use condoms, respectively.

Married women in the priority population experiencing IPV had non-significantly higher odds (age-adjusted OR = 1.23, 95% CI: 0.96–1.60; *p* = 0.11) of being motivated to use male condoms compared to women not experiencing IPV. Amongst women motivated to use condoms, those experiencing IPV did not differ in having access to condoms from women not experiencing IPV (*p* = 0.16). Amongst women who were motivated to use male condoms and could access them, no difference in condom use at last sex was found between those reporting and not reporting IPV (*p* = 0.66).

## Discussion

HIV prevention cascades have been proposed as a tool for identifying appropriate targeted interventions for priority populations who could benefit from HIV prevention methods but have low use of these methods [36]. In earlier studies [37, 38] – and in the current study of HIV-uninfected married women in Manicaland – women experiencing IPV have high risk of HIV acquisition and therefore represent a priority population who could benefit from prevention methods. To our knowledge, this is the first study to measure an HIV prevention cascade for married women at risk of HIV acquisition and subject to IPV and to investigate differences with the cascade for other married women at risk.

In Manicaland, uninfected married women experiencing IPV had 2.26-times greater odds than other married women of being at risk of acquiring HIV infection and of potentially benefiting from HIV prevention methods. 20.7% (300/1449) of the priority population of married women for use of HIV prevention methods, based on their own or their partner’s sexual risk-behaviours, had experienced sexual or physical violence in the previous 12 months. No differences were found between the main bars in the condom cascades for married women in the priority population who experienced IPV and those who did not. However, in both groups, few women used condoms, and the largest gaps in the cascade were in motivation to use and in capacity to use condoms effectively in women who were motivated and able to access them. In each group, the most common barriers to condom use were low HIV risk perception, limited knowledge about condoms, and condom use in marriage stigma (motivation barriers), lack of acceptable provision (access barrier), and male partner opposition and weak negotiating skills (barriers to capacity to use effectively). Importantly, married women experiencing IPV who were motivated to use condoms and able to access them were more likely than those not reporting IPV to report male partner opposition as a barrier to their using condoms.

IPV prevalence in our study is high and in line with an earlier study on gender-based violence in Manicaland [51]. It is somewhat higher though than reported for Manicaland in a national survey in 2015/16 (physical violence: 19.2% vs. 14.5%; sexual violence: 8.7% vs. 7.5%) [28]. This may be due to differences between the women included in the analyses (currently married women aged 15–54 in this study; ever-married women aged 15–49 in the national survey). However, the women’s sociodemographic characteristics associated with experiencing IPV in Manicaland were consistent with those reported in other settings. For example, previous studies have also found that IPV is more common in younger women [39, 40] and in less educated women [7, 41].

The findings from this analysis indicate that, to further reduce HIV incidence in married women in Manicaland, IPV interventions, supplemented with additional HIV control measures, will be needed both to reduce levels of men’s and women’s behaviours associated with HIV acquisition risk and to increase partner’s use of male condoms and other efficacious HIV prevention methods in those who continue to be at risk. The association we found between IPV and behaviours associated with HIV acquisition risk probably reflects a combination of a causal link between IPV and HIV risk-behaviour, reverse causality, and unconnected pathways arising from overlapping structural determinants (i.e. gender and economic inequalities). Suffering IPV can cause women to seek social support and may result in their engaging in new sexual relationships, having an extra-marital sexual relationship, drinking alcohol, and having a spouse with other partners or a sexually transmitted infection (particularly if such spouses are more inclined to become violent) all seem plausible circumstances that could lead to IPV. Interventions that reduce IPV, therefore, may have only a partial effect in reducing married women’s exposure to behaviours associated with HIV acquisition risk. This interpretation is consistent with the mixed findings from IPV intervention trials in African populations. In the IMAGE Trial in South Africa, a structural intervention combining microfinance with gender awareness and HIV education failed to reduce behaviours associated with HIV acquisition risk and HIV incidence [24]; whilst, in the SASA! Trial in Uganda, community mobilisation, that included female and male community activists, reduced male sexual concurrency by 43% [42].

Previous studies found that male dominance and cultural norms are associated with low condom use in married couples [43]. In the HIV prevention cascades for married women at risk of HIV infection, we found a large gap in capacity to use condoms effectively and that male partner resistance plays a large part in explaining this gap particularly when IPV is present in the relationship. This finding suggests that IPV interventions, like the MAISHA intervention in Tanzania, which promote healthy relationships and empower women to negotiate non-violent ways to resolve conflicts [44], could contribute to reducing HIV incidence by helping both to increase condom use (even when IPV is still present) and to reduce behaviours associated with HIV acquisition risk. However, we found no differences in the gaps between the main bars of the condom cascade for married women at risk of HIV reporting and not reporting IPV in Manicaland and, apart from the difference in male partner resistance, the explanatory barriers were similar.

These findings suggest that IPV interventions, on their own, may not be effective in reducing HIV risk in married women in African populations. To achieve this, IPV interventions may need to be combined with individual-level interventions (e.g. to improve risk perception [45]), further structural interventions (e.g. addressing social norms against condom use in marriage and making condom provision for married women more acceptable), and additional activities to reduce underlying behaviours associated with HIV risk. As many married women not experiencing IPV could also benefit from HIV prevention methods and report similar cascade gaps and explanatory barriers to reducing HIV risk, these interventions would need to support all married women at risk. Many of these elements were included in the comprehensive SHARE intervention in Rakai, Uganda, which reduced both IPV (physical and sexual) and HIV incidence [46].

The strengths of this study include the research settings, that have coinciding high levels of IPV and HIV prevalence, a large representative general population sample, and unique data that include both the UNAIDS recommended questions on IPV (Joint United Nations Programme on HIV/AIDS, 2020) and bespoke questions for measuring a published HIV prevention cascade framework. The main limitations are use of cross-sectional data (limiting our ability to draw causal inferences), exclusion of unmarried women, exclusion of HIV prevention methods other than male condoms, and reliance on self-reported data for the variables on IPV, behaviours associated with HIV risk, and condom use. We focused on married and cohabiting women because interventions could be more feasible in this group in which the dynamics of sustained intimate partner interactions may influence outcomes more predictably over time. Male condoms were investigated because these were the main HIV prevention method used during the survey period. Since the survey, the Zimbabwe government has scaled-up oral pre-exposure prophylaxis (PrEP) and approved injectable PrEP. For married women who want to use PrEP and can access it, lack of partner support may be a smaller barrier to effective use than for male condoms as it can be a female-controlled method and doesn’t control fertility. However, fear of pain may be a barrier to motivation to use injectable PrEP [52, 53]. Future studies should explore these new prevention methods through the lens of the HIV prevention cascade to better understand the gaps in motivation, access and effective use in married and unmarried women at HIV risk comparing those experiencing and not experiencing IPV.

## Conclusion

We conducted the first HIV prevention cascade analysis on the influence of IPV on exposure to HIV infection and explanatory barriers to use of HIV prevention methods in married women in an African population with hyper-endemic HIV prevalence. The findings reinforce the need for integrated IPV/HIV interventions to include activities that address gender-based economic and social inequalities and help women to strengthen their negotiating skills. However, for these interventions to have a greater impact in reducing overall HIV risk in married women, they must be extended to support those not recently experiencing IPV and to address other important barriers to reducing behaviours associated with HIV risk and increasing effective use of HIV prevention methods. These barriers include lack of risk perception, social norms on condom use in marriage, and lack of acceptable service provision.

## Electronic Supplementary Material

Below is the link to the electronic supplementary material.

Supplementary Material 1

## Data Availability

Due to the sensitive nature of data collected, including information on HIV status, treatment and sexual risk behaviour, the Manicaland Centre for Public Health does not make full analysis datasets publicly available. Summary datasets of household and background sociodemographic individual questionnaire data, covering rounds 1-8 (1998-2021), are publicly available and can be downloaded from the Manicaland Centre for Public Health website: http://www.manicalandhivproject.org/data-access.html. Quantitative data used for analyses produced by the Manicaland Centre for Public Health are available upon request following completion of a data access request form found here: http://www.manicalandhivproject.org/data-access.html, or by emailing Professor Simon Gregson at sajgregson@aol.com. Additionally, summary HIV incidence and mortality data spanning rounds 1-6 (1998-2013), created in collaboration with the ALPHA Network are available via the DataFirst Repository here: https://www.datafirst.uct.ac.za/dataportal/index.php/catalog/ALPHA/about.
