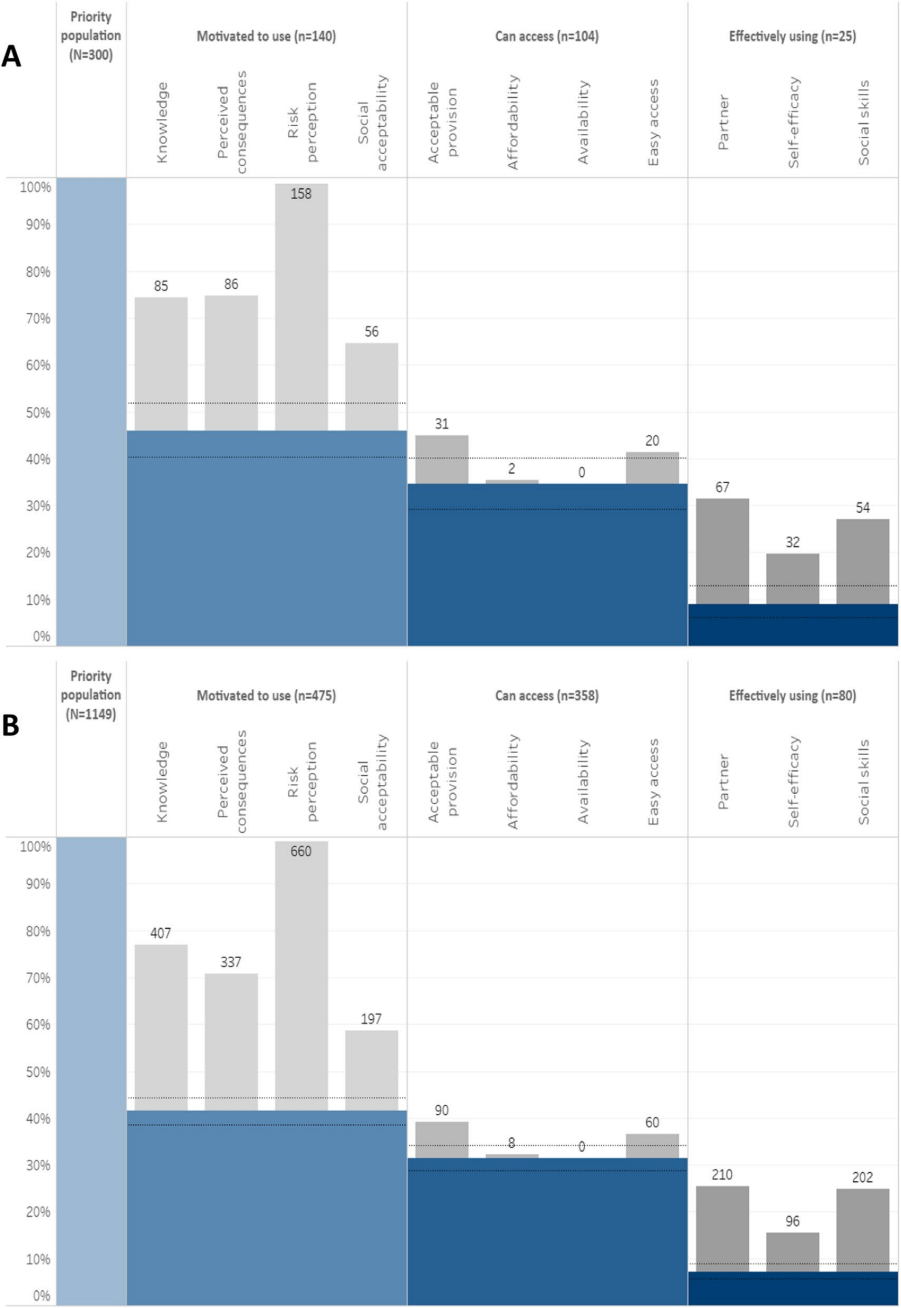# Correction: Intimate partner violence, behaviours associated with risk of HIV acquisition and condom use in married women in Manicaland, East Zimbabwe: An HIV prevention cascade analysis

**DOI:** 10.1186/s12905-024-03466-5

**Published:** 2024-11-29

**Authors:** Alexandra A. Cordeiro, Louisa Moorhouse, Tawanda Dadirai, Rufurwokuda Maswera, Angela Y. Chang, Constance Nyamukapa, Simon Gregson

**Affiliations:** 1 MRC Centre for Global Infectious Disease Analysis, Department of Infectious Disease Epidemiology, School of Public Health, Imperial College London, Level 2, Faculty Building South Kensington Campus, London, SW7 2AZ UK; 2 Biomedical Research and Training Institute, 10 Seagrave Avondale, Harare, Zimbabwe; 3 Danish Institute for Advanced Study, University of Southern Denmark, Fioniavej 34, Odense, 5230 Denmark; 4 Department of Clinical Research, University of Southern Denmark, J.B. Winsløws Vej 19,3, Odense, 5000 Denmark

**Correction** **:** **BMC Women's Health 24, 592 (2024)**

**https://doi.org/10.1186/s12905-024-03428-x**

Following the publication of the original article [1], it was discovered that Panel B in Figure 2 was omitted. The corrected Figure 2 is presented below.

Incorrect Fig. 2


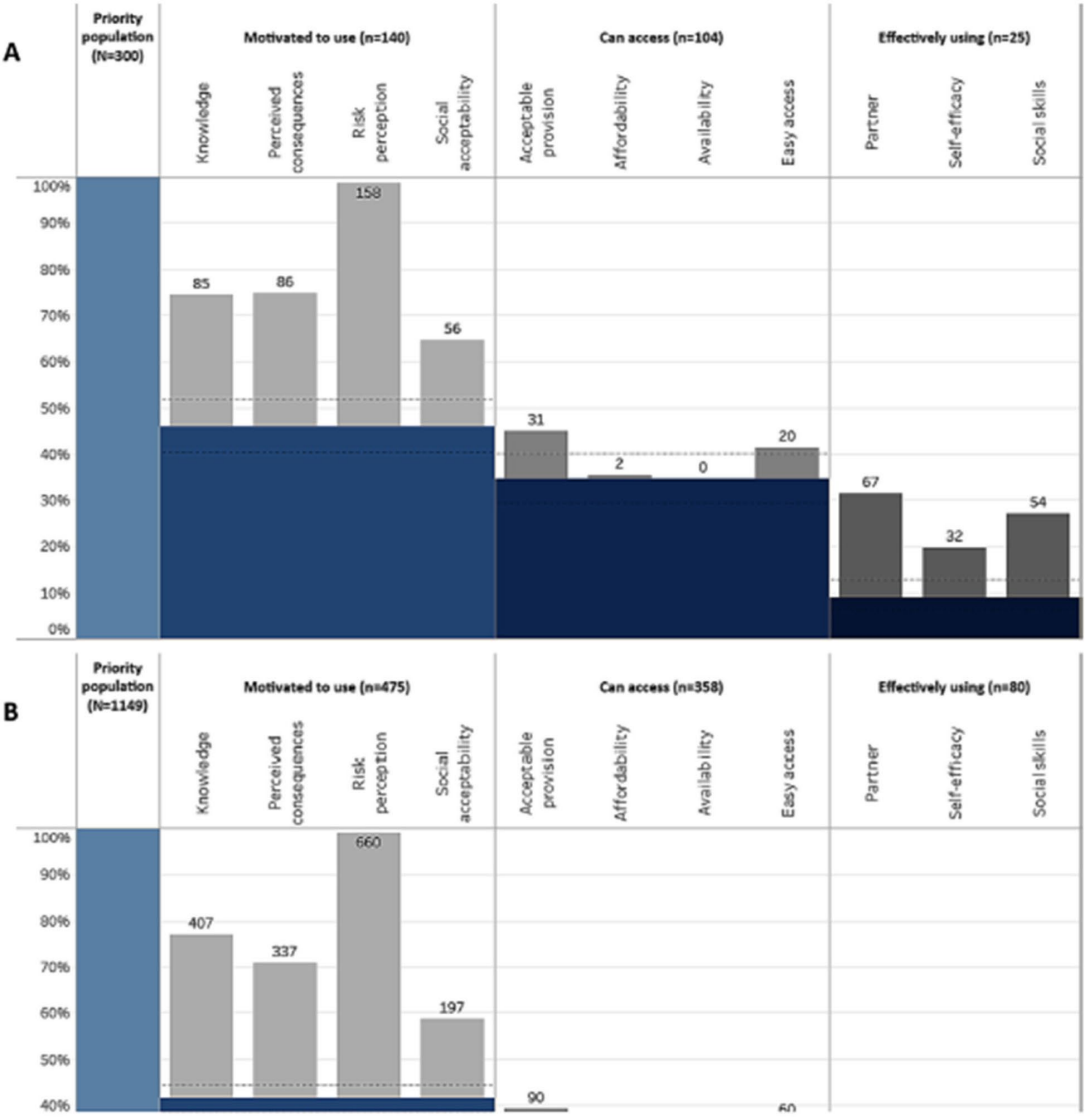


Correct Fig. 2